# Complete genome sequence of *Arthrobacter* sp. PAMC25564 and its comparative genome analysis for elucidating the role of CAZymes in cold adaptation

**DOI:** 10.1186/s12864-021-07734-8

**Published:** 2021-06-02

**Authors:** So-Ra Han, Byeollee Kim, Jong Hwa Jang, Hyun Park, Tae-Jin Oh

**Affiliations:** 1 Department of Life Science and Biochemical Engineering, Graduate School, SunMoon University, 70 Sunmoon-ro 221, Tangjeong-myeon, 31460 Asan-si, Chungnam, Republic of Korea; 2 Department of Dental Hygiene, College of Health Science, Dankook University, 119 Dandae-ro, Dongnam-gu, 31116 Cheonan-si, Chungnam, Republic of Korea; 3 Division of Biotechnology, College of Life Science and Biotechnology, Korea University, 02841 Seoul, Republic of Korea; 4 Genome-based BioIT Convergence Institute, 70 Sunmoon-ro 221, Tangjeong-myeon, 31460 Asan-si, Chungnam, Republic of Korea; 5 Department of Pharmaceutical Engineering and Biotechnology, SunMoon University, 70 Sunmoon-ro 221, Tangjeong-myeon, 31460 Asan-si, Chungnam, Republic of Korea

**Keywords:** *Arthrobacter* species, CAZyme, Cold-adapted bacteria, Genetic patterns, Glycogen metabolism, Trehalose pathway

## Abstract

**Background:**

The *Arthrobacter* group is a known set of bacteria from cold regions, the species of which are highly likely to play diverse roles at low temperatures. However, their survival mechanisms in cold regions such as Antarctica are not yet fully understood. In this study, we compared the genomes of 16 strains within the *Arthrobacter* group, including strain PAMC25564, to identify genomic features that help it to survive in the cold environment.

**Results:**

Using 16 S rRNA sequence analysis, we found and identified a species of *Arthrobacter* isolated from cryoconite. We designated it as strain PAMC25564 and elucidated its complete genome sequence. The genome of PAMC25564 is composed of a circular chromosome of 4,170,970 bp with a GC content of 66.74 % and is predicted to include 3,829 genes of which 3,613 are protein coding, 147 are pseudogenes, 15 are rRNA coding, and 51 are tRNA coding. In addition, we provide insight into the redundancy of the genes using comparative genomics and suggest that PAMC25564 has glycogen and trehalose metabolism pathways (biosynthesis and degradation) associated with carbohydrate active enzyme (CAZymes). We also explain how the PAMC26654 produces energy in an extreme environment, wherein it utilizes polysaccharide or carbohydrate degradation as a source of energy. The genetic pattern analysis of CAZymes in cold-adapted bacteria can help to determine how they adapt and survive in such environments.

**Conclusions:**

We have characterized the complete *Arthrobacter* sp. PAMC25564 genome and used comparative analysis to provide insight into the redundancy of its CAZymes for potential cold adaptation. This provides a foundation to understanding how the *Arthrobacter* strain produces energy in an extreme environment, which is by way of CAZymes, consistent with reports on the use of these specialized enzymes in cold environments. Knowledge of glycogen metabolism and cold adaptation mechanisms in *Arthrobacter* species may promote in-depth research and subsequent application in low-temperature biotechnology.

**Supplementary Information:**

The online version contains supplementary material available at 10.1186/s12864-021-07734-8.

## Background

The *Arthrobacter* genus is a member of the family *Micrococcaceae*, which belongs to the phylum *Actinobacteria* [1, 2]. *Arthrobacter* species are often isolated from soil, where they contribute to biochemical cycles and decontamination [3]. Additionally, these species have been isolated worldwide from a variety of environments, including sediments [4], human clinical specimens [5], water [6], glacier cryoconite [7], sewage [8], and glacier ice [9]. Cold environments represent about 75 % of the earth, and their study provides information about new microorganisms and their evolution in cold environments [10]. Psychrophilic microorganisms have colonized all permanently cold environments, from the deep sea to mountains and polar regions [11]. Cold-adapted microorganisms utilize a wide range of metabolic strategies to grow in diverse environments. In general, the ability to adapt to low temperatures requires that microorganisms sense a decrease in temperature, which induces upregulation of cold-associated genes [12]. *Arthrobacter* is a gram-positive obligate aerobe that requires oxygen to grow in a variety of environments. Obligate aerobes grow through cellular respiration and use oxygen to metabolize substances like sugars, carbohydrates, or fat to obtain energy [13, 14]. However, there is a still lack of research on how obligate aerobes acquire adequate energy in cold environments.

Carbohydrate active enzymes (CAZymes) have functions associated with biosynthesis, binding, and catabolism of carbohydrates. This classification system is based on amino acid sequence similarity, protein folds, and enzymatic mechanism. Thus, one can understand overall enzyme function and carbohydrate metabolism through CAZymes [15]. These enzymes are classified based on their catalytic activity: glycoside hydrolase (GH), carbohydrate esterase (CE), polysaccharide lyase (PL), glycosyltransferase (GT), and auxiliary activity (AA). In addition, CAZymes may have non-catalytic subunits like a carbohydrate-binding module (CBM). CAZymes are already well known in biotechnology, and their industrial applications are of interest to many researchers because they produce precursors for bio-based products such as food, paper, textiles, animal feed, and various chemicals, including biofuels [16, 17].

Most bacteria can use glycogen as an energy storage compound, and the enzymes involved in its metabolism are well known. A recent study showed the physiological impact of glycogen metabolism on the survival of bacteria living in extreme environments [18]. Some microorganisms can adapt quickly to continuously changing environmental conditions by accumulating energy storage compounds to cope with transient starvation periods. These strategies use glycogen-like structures such as polysaccharides composed of α-_D_-glycosyl units connected by α-1,4 linkages and branched by α-1,6 glycosidic linkages. Such biopolymers differ in their chain length and branching occurrence. To be used as carbon and energy sources, their glucose units are released by specific enzymes [19].

Microorganisms have synergistic enzymes capable of decomposing plant cell walls to release glucose. This phenomenon can be used for energy supply to maintain microbial growth [20]. Starch is an excellent source of carbon and energy for microbes that produce proteins responsible for extracellular hydrolysis of starch, in-cell absorption of fructose, and further decomposition into glucose [21]. In addition, strains that metabolize glycogen show important physiological functions, including use of energy storage compounds for glycogen metabolism. These pathways act as carbon pools that regulate carbon fluxes [22], and partly, this ability is attributed to CAZymes. Using comparative genome analysis of bacteria isolated from cold environments and the genetic patterns of CAZymes within them, this study provides an understanding of how survival adaptation can be achieved in extremely low-temperature environments.

## Results and discussion

### Profile of the complete genome of Arthrobacter sp. PAMC25564

**Fig. 1:**
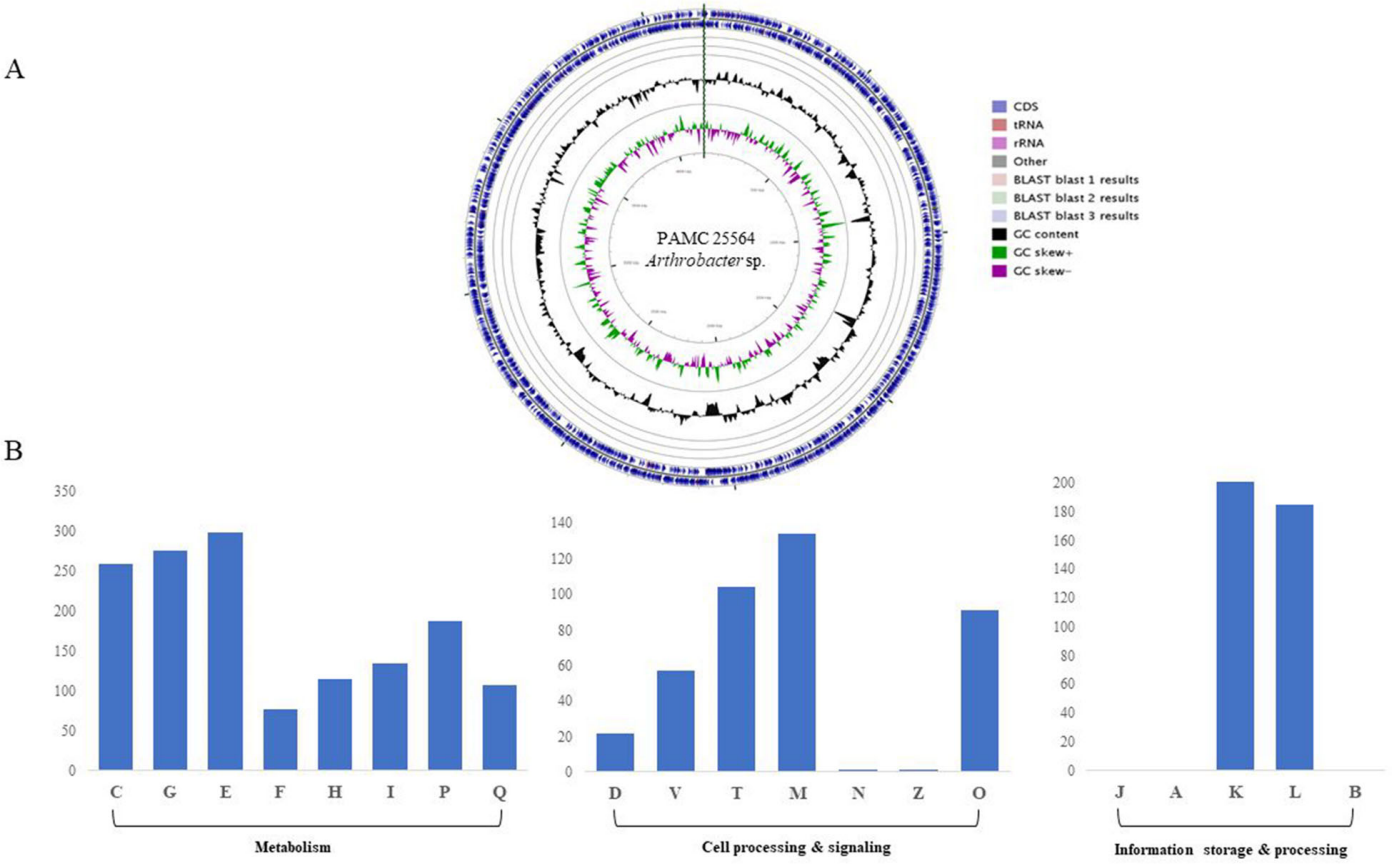
** A**. Circular map of *Arthrobacter* sp. PAMC25564 genome, **B**. COG functional categories for forward coding sequences. Metabolism: C, energy production and conversion; G, carbohydrate transport and metabolism; E, amino acid transport and metabolism; F, nucleotide transport and metabolism; H, coenzyme transport and metabolism; I, lipid transport and metabolism; P, inorganic ion transport and metabolism; and Q, secondary metabolites biosynthesis, transport, and catabolism. Cell processing and signaling: D, cell cycle control, cell division, and chromosome partitioning; V, defense mechanisms; T, signal transduction mechanisms; M, cell wall/membrane/envelope biogenesis; N, cell motility; Z, mobilome, prophages, and transposons; and O, posttranslational modification, protein turnover, and chaperones. Information storage and processing: J, translation, ribosomal structure, and biogenesis; A, RNA processing and modification; K, transcription; L, replication, recombination and repair; and B, chromatin structure and dynamics

As shown in Table 1, the complete genome of *Arthrobacter* sp. PAMC25564 is composed of a circular chromosome of 4,170,970 bp with a 66.74 % GC content. 3,829 genes were predicted on the chromosome of which 3,613 protein-encoding genes were functionally assigned, whereas the remaining genes were predicted as hypothetical proteins. We annotated 147 pseudogenes, 15 rRNA genes, and 51 tRNA genes distributed through the genome. From the predicted genes, 3,449 (90.08 %) were classified into 20 functional Clusters of Orthologous Groups (COG) categories, whereas the remaining 380 (9.92 %) remained un-classified. The most numerous COG categories were S genes with unknown function (705 genes), transcription (category K, 298 genes), amino acid transport and metabolism (category E, 280 genes), carbohydrate transport and metabolism (category G, 276 genes), and energy production and conversion (category C, 259 genes) (Fig. 1). Many of these genes are related to amino acid transport, transcription, carbohydrate transport, and energy production/conversion, which suggests that this strain utilizes CAZymes for energy storage and carbohydrate metabolism. Most bacteria rely on cell respiration to catabolize carbohydrates to obtain the energy used during photosynthesis for converting carbon dioxide into carbohydrates. The energy is stored temporarily in the form of high-energy molecules such as ATP and used in several cell processes [23, 24]. However, *Arthrobacter* is already known as a genus of bacteria that is commonly found in cold environments. All species in this genus are gram-positive obligate aerobes and as such require oxygen to grow. These organisms use oxygen to metabolize substances like sugars, polysaccharides, or fats, to obtain energy as cellular respiration [14]. Therefore, we predicted that the PAMC25564 strain could also utilize carbohydrate degradation to obtain energy through these results.

**Table 1:** Genome features of *Arthrobacter* sp. PAMC25564

Feature	Value
**A; Genome Statistics**	
Contigs	1
Total length bp;	4,170,970
N50	4,170,970
L50	1
GC %;	66.74
**B; Genome features**	
Assembly level	Complete genome
Chromosome genes	3,829
Protein-coding genes	3,613
Pseudogenes	147
rRNA genes	15
tRNA genes	51

### 16 S rRNA phylogenetic analysis and average nucleotide identity (ANI) values

**Fig. 2:**
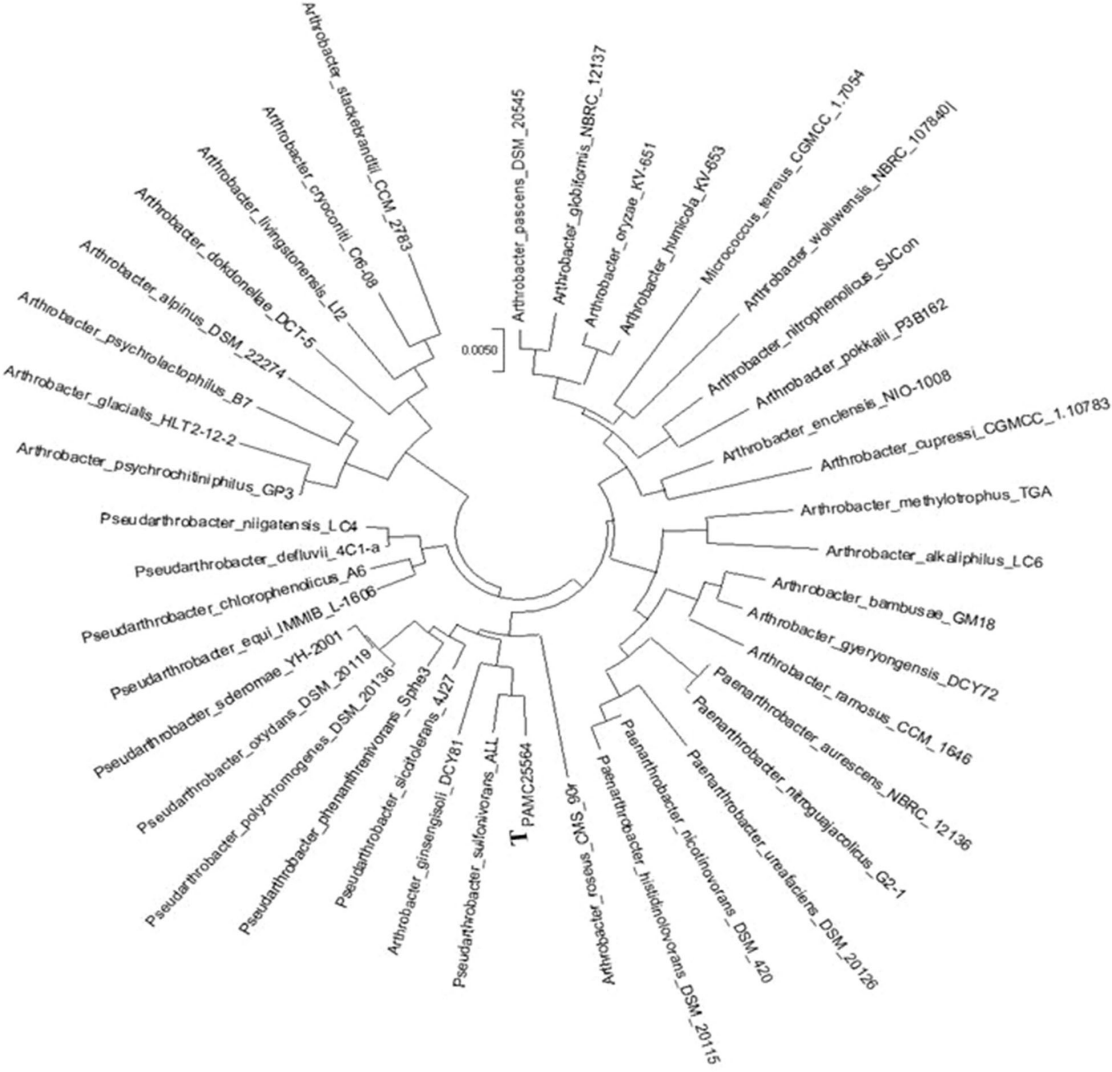
Phylogenetic tree of *Arthrobacter sp.* PAMC25564. The phylogenetic tree was generated using the Maximum Likelihood method and Tamura-Nei model in MEGA X, based on 16 S rRNA sequences. This study used the 16 S rRNA sequence of 40 strains. The tree shows the relationship between three *Arthrobacter* strains and six *Pseudarthrobacter* strains and their phylogenetic positions as compared with that of our strain. The capital T in bold indicates our strain

The identification of *A.* sp. PAMC25564 was verified using 16 S rRNA sequence analysis (Fig. 2). This strain is phylogenetically placed among *Arthrobacter* and *Pseudarthrobacter* species. The results from phylogenetic analysis, Basic Local Alignment Search Tool, and EzBio Cloud revealed closely related strains such as *P. sulfonivirans* ALL (T) (99.09 %), *P. siccitolerans* 4J27 (T) (98.48 %), *A. ginsengisoli* DCY81 (T) (98.23 %), and *P. phenanthrenivorans* Sphe3 (T) (98.13 %). These results confirmed that isolate PAMC25564 belongs to the family *Micrococcaceae*, phylum *Actinobacteria*. Recently, several *Arthrobacter* species have been reclassified into new genera, based on 16 S rRNA sequence similarities and chemotaxonomic traits such as peptidoglycan types, quinone systems, and/or polar lipid profiles [25]. It has been proposed to reclassify five genera within the genus *Arthrobacter*: *Paenarthrobacter* gen. nov., *Pseudarthrobacter* gen. nov., *Glutamicibacter* gen. nov., *Paeniglutamicibacter* gen. nov., and *Pseudoglutamicibacter* gen. nov. Among them, the genus *Arthrobacter* would be reclassified into the *Pseudarthrobacter* group as: *A. chlorophenolicus*, *A. defluvii*, *A*. *equi*, *A*. *niigatensis*, *A*. *oxydans*, *A*. *phenanthrenivorans*, *A*. *polychromogenes*, *A*. *scleromae*, *A*. *siccitolerans*, and *A*. *sulfonivorans* [26].

**Fig. 3:**
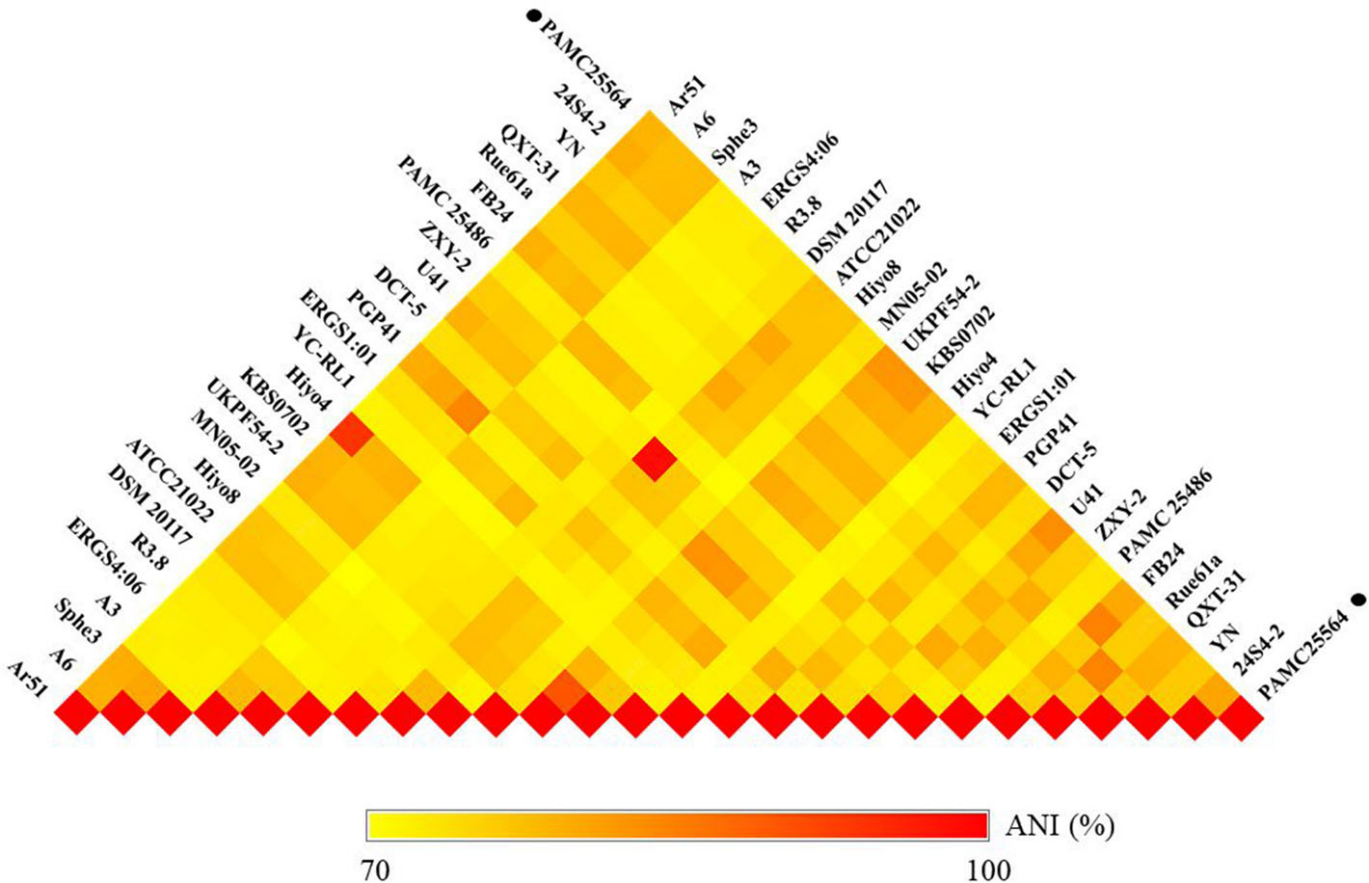
Orthologous Average Nucleotide Identity of *Arthrobacter* sp. PAMC 25564 and 25 other genomes calculated using OrthoANI. ANI results are colored yellow to red according to their value mean from 70 to 100 %. They were calculated using an OrthoANI in OAT (Orthologous Average Nucleotide Identity Tool). Strains belonging to the same species are marked with strong color. *Arthrobacter* sp.: PAMC25564 (NZ_CP039290.1), 24S4-2 (NZ_CP040018.1), YN (NZ_CP022436.1), QXT-31 (NZ_CP019304.1), Rue61a (NC_018531.1/CP003203.1), FB24 (NC_008541.1/CP000454.1), PAMC25486 (NZ_CP007595.1), ZXY-2 (NZ_CP017421.1), U41 (NZ_CP015732.1), DCT-5 (NZ_CP029642.1), PGP41 (NZ_CP026514.1), ERGS1:01 (NZ_CP012479.1), YC-RL1 (NZ_CP013297.1), Hiyo4 (AP014718.1), KBS0702 (NZ_CP042172.1), UKPF54-2 (NZ_CP040174.1), MN05-02 (AP018697.1), Hiyo8 (AP014719.1), and ATCC21022 (NZ_CP014196.1); *Arthrobacter crystallopoietes*: DSM 20117 (NZ_CP018863.1); *Arthrobacter alpinus*: R3.8 (NZ_CP012677.1), ERGS4:06 (NZ_CP013200.1), and A3 (NZ_CP013745.1); *Pseudarthrobacter phenanthrenivorans*: Sphe3 (NC_015145.1/CP002379.1); *Pseudarthrobacter chlorophenolicus*: A6 (NC_011886.1/CP001341.1); and *Pseudarthrobacter sulfonivorans*: Ar51 (NZ_CP013747.1). The black circle indicates our strain

In general, bacterial comparative genome analysis uses the ANI methods. As shown in Fig. 3, each ANI value ranged from 70.67 to 98.46 %. So we see that comparative genome results are much lower than the common ANI values of 92–94. The ANI analysis shows the average nucleotide identity of all bacterial orthologous genes that are shared between any two genomes and offers a robust resolution between bacterial strains of the same or closely related species (i.e., species showing 80–100 % ANI) [27]. However, ANI values do not represent genome evolution, because orthologous genes can vary widely between the genomes being compared. Nevertheless, ANI closely reflects the traditional microbiological concept of DNA-DNA hybridization relatedness for defining species, so many researchers use this method, since it takes into account the fluid nature of the bacterial gene pool and hence implicitly considers shared functions [28]. The results mean the PAMC25564 strain could either belong to the species from which *Arthrobacter* diverged, or could be a *Pseudarthrobacter* closely related new species. However, this study classified the strain and allocated a species through 16 S rRNA sequencing and ANI. While our classification is not conclusive, the PAMC25564 strain will probably be reclassified into the genus *Pseudarthrobacter* in a follow-up study.

### CAZyme-encoding genes in Arthrobacter sp. PAMC25564

Among the 3,613 identified protein-encoding genes in PAMC25564, 108 were significantly annotated and classified into CAZyme groups (GH, GT, CE, AA, CBM, and PL) using dbCAN2. The results provided an insight into the carbohydrate utilization mechanisms of PAMC25564. Signal peptides gene retention predicted that 11 genes contained in CAZyme of strain PAMC26554 through Signal P tool. We found that proteins were distributed as follows: 33 GHs, 45 GTs, 23 CEs, 5 AAs, and 2 CBMs. However, no protein was assigned to the PL group. The GH gene annotations revealed that the PAMC25564 genome contains genes involved in glycogen and trehalose metabolism pathways such as β-glucosidase (GH1), glycogen debranching proteins (CBM48 and GH13_11), (1→4)-α-D-glucan 1-α-D-glucosylmutase (GH13_26), α-glucosyltransferase (GH13), α-trehalose phosphorylase (GH65), and 4-α-glucanotransferase (GH77) (Table 2). Previous studies showed the complex interplay of glycogen metabolism in colony development of *Streptomycetes* (in *Actinomycetes* species was only reported), showing that spore germination is followed by an increase in glycogen metabolism [29]. The underlying genetic and physiological mechanisms of spore germination remain unknown, but some mechanisms associated with the accumulation of nutrients such as biomass and storage materials in the substrate mycelium during morphological phases of development have been reported [30]. However, not much research has been done yet about whether gram-positive obligate aerobes have glycogen metabolism mechanisms. Recently, *Shigella* sp. PAMC28760, a pathogen isolated from Antarctica, was also reported to be able to adapt and survive in cold environments through glycogen metabolism [31]. Also, *Bacillus* sp. TK-2 has been reported to possess cold evolution adaptability through CAZyme genes related to degradation of polysaccharides including cellulose and hemicellulose [32]. These complete genome analyses uncover genomic information and evolutionary insights regarding diverse strains and species from cold environments. However, characteristics of glycogen metabolism in prokaryotes remain less well-studied than those in eukaryotes, and the metabolism of microorganisms isolated from cold environments are not well understood [33]. This study predicts the role of CAZymes in cold adaptation, specifically as being those PAMC25564 genes involved in glycogen and trehalose metabolism.

**Table 2:** List of CAZyme GH enzymes from *Arthrobacter* sp. PAMC25564

CAZyme group	Enzyme activity	Gene position	EC number	Number
GH1	β-Glucosidase	1206507_1208054	EC 3.2.1.21	2
		1548357_1546930		
GH2	β-Glucuronidase	230633_228825	EC 3.2.1.31	1
GH3	β-Glycosyl hydrolase	226049_223737	-	2
		1615559_1617091		
GH4	6-Phospho-β-glucosidase	1608453_1606978	EC 3.2.1.86	1
GH13	Malto-oligosyltrehalose/trehalohydrolaseGH13_10;	1599543_1601333	EC 3.2.1.141	8
	Limit dextrin α-1,6-maltotetraose-hydrolase GH13_11;	4158486_4156372	EC 3.2.1.196	
	Trehalose synthase α-Amylase GH13_16;	4150667_4148871	EC 5.4.99.16 EC 3.2.1.1	
	Malto-oligosyltrehalose synthase GH13_26;	1597186_1599498	EC 5.4.99.15	
	Glucanase glge GH13_3;	4152718_4150673	EC 3.2.1.-	
	α-Glucosidase GH13_30;	1490553_1492259	EC 3.2.1.20	
	Limit dextrin α-1,6-maltotetraose-hydrolase CBM48 + GH13_11;	1594748_1597189	EC 3.2.1.196	
	1,4-α-Glucan glycogen; branching enzyme CBM48 + GH13_9;	4148869_4145174	EC 2.4.1.18	
GH15	Glucoamylase	2725735_2726796	EC 3.2.1.3	4
		1210063_1211832		
		2262201_2264033		
	Trehalose-6-phosphate phosphatase	943180_945807	EC 3.1.3.12	
GH23	Peptidoglycan-binding lysm	3355397_3356797	-	2
	Membrane-bound lytic murein transglycosylase	3043759_3043151	EC 4.2.2.-	
GH25	1,4-β-N-Acetylmuramidase	1863081_1865612	EC 3.2.1.92 -	1
GH30	Endo-1,6-β-galactosidase	731722_733167	EC 3.2.1.164	1
GH32	Sucrose-6-phosphate hydrolase	3442058_3440550	EC 3.2.1.26	2
	β-Fructosidase	1383596_1384843	EC 3.2.1.26	
GH33	Sialidase	613011_614603	EC 3.2.1.18	1
GH38	α-Mannosidase	4082745_4079713	EC 3.2.1.24	1
GH53	Galactosidase	2955779_2956930	-	1
GH65	Maltose phosphorylase/ Trehalose phosphorylase	392560_390227	EC 2.4.1.8 EC 2.4.1.64	2
	Trehalose-6-phosphate phosphatase	342376_339176	EC 3.1.3.12	
GH76	Fructose-bisphosphate aldolase	137645_138862	EC 4.1.2.13	1
GH77	4-α-Glucanotransferase amylomaltase;	2730522_2728363	EC 2.4.1.25	1
GH109	Gluconokinase	787401_788513	EC 2.7.1.12	2
		236269_235103		

### Comparison of Arthrobacter sp. PAMC25564 genome characteristics with those from closely related species

**Fig. 4:**
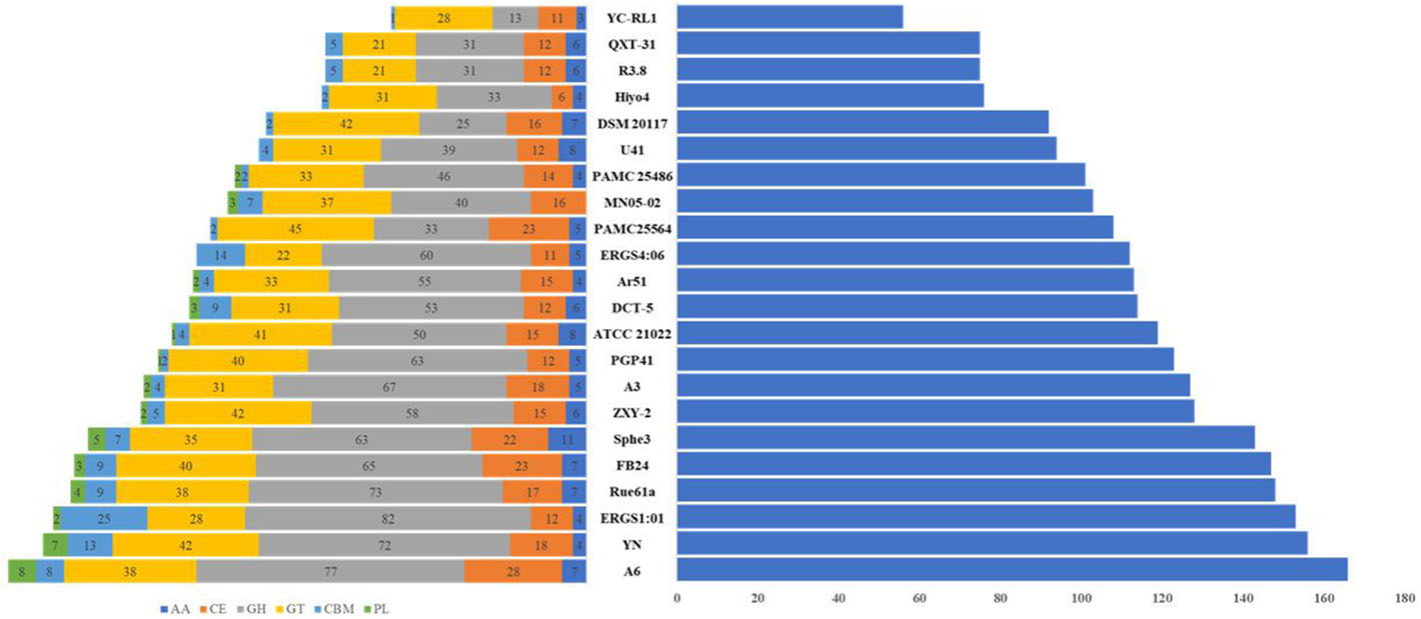
Comparative CAZyme-encoding genes found in the genome of *Arthrobacter* species. On the left side is the CAZyme family and on the right side is the total CAZyme number: GT, glycosyltransferase; GH, glycoside hydrolase; CE, carbohydrate esterase; CBM, carbohydrate-binding modules; and AA, auxiliary activity. The CAZyme family is colored, as indicated below the figure. *Arthrobacter* sp.: PAMC25564, 24S4-2, YN, QXT-31, Rue61a, FB24, PAMC25486, ZXY-2, U41, DCT-5, PGP41, ERGS1:01, YC-RL1, Hiyo4, KBS0702, UKPF54-2, MN05-02, Hiyo8, and ATCC21022 *Arthrobacter crystallopoietes*: DSM 20117 *Arthrobacter alpinus*: R3.8, ERGS4:06, and A3 *Pseudarthrobacter phenanthrenivorans*: Sphe3 *Pseudarthrobacter chlorophenolicus*: A6 and *Pseudarthrobacter sulfonivorans*: Ar51

We compared CAZyme genes from *Arthrobacter* species to speculate about their bacterial lifestyles and identified relevant CAZymes for potential applications in biotechnology. Considering the accessibility of available genome data, the complete genomes of 26 strains were chosen for the comparative analysis of CAZymes: 19 genomes of *Arthrobacter* spp., one genome of *A*. *crystallopoietes*, three genomes of *A*. *alpinus*, and three genomes of *Pseudarthrobacter* spp. (Table 3). Our results showed that the number of total CAZymes in each genome ranged from a minimum of 56 (*A*. sp. YC-RL1) to a maximum of 166 (*P. chlorophenolicus* A6) (Fig. 4). We predicted that common CAZyme genes such as CE14, CE9, GH23, GH65, GT2, GT20, GT28, GT39, GT4, and GT51 would appear in each of the 26 genomes. However, when we compared strains isolated from cold environments, we found CAZyme genes were more common than what is found in the 26 comparison genomes. They include CE1, CE4, CE9, CE10, CE14, AA3, AA7, CBM48, GH1, GH3, GH13, GH15, GH23, GH25, GH38, GH65, GH76, GT2, GT4, GT20, GT28, GT39, and GT51. In particular, CAZyme members GH13, GH65, GH77, GT5, and GT20 (glycogen and trehalose-related genes) are involved in energy storage. This study focuses on those genes related to adaptations in metabolism that allow the species to withstand cold environments. These genes are involved in glycogen degradation and trehalose pathways and were found in strains PAMC25564, 24S4-2, FB24, Hiyo8, KBS0702, MN05-02, PGP41, QXT-31, U41, UKPF54-2, A6, Ar51, and sphe3. These *Arthrobacter* species isolated from extreme environments have a family of CAZymes and the related genes for proteins with a strong ability to store and release energy and this permits them to survive in such cold areas. We found that strain PAMC25564 had the largest number of CAZyme genes related to glycogen metabolism and the trehalose pathway. In general, CAZymes are a large group of proteins that are mainly responsible for the degradation and biosynthesis/modification of polysaccharides but not all the members of this group are secreted proteins. This study confirms small differences in the gene pattern of CAZymes between species (Additional file 1: Figure S1).

**Table 3:** Genome information of 26 strains including *Arthrobacter* sp. PAMC25564

Species	Strain	Size Mb;	GC %;	Replicons	Plasmid	Gene	Protein	tRNAs	rRNAs	References
*Arthrobacter* sp.	PAMC25564	4.17097	66.70	NZ_CP039290.1	0	3,829	3,613	51	15	**This study**
	24S4-2	5.56375	65.10	NZ_CP040018.1	0	5,152	4,522	50	15	Unpublished
	YN	5.06355	62.70	NZ_CP022436.1	0	4,673	4,387	55	18	Unpublished
	QXT-31	5.04157	66.00	NZ_CP019304.1	0	4,593	4,379	54	18	Unpublished
	Rue61a	5.08104	62.23	NC_018531.1/CP003203.1	2	4,693	4,568	53	18	[34]
	FB24	5.07048	65.42	NC_008541.1/CP000454.1	3	4,623	4,486	51	15	[35]
	PAMC25486	4.59358	62.80	NZ_CP007595.1	0	4,154	3,995	53	18	Unpublished
	ZXY-2	5.05871	63.35	NZ_CP017421.1	5	4,700	4,505	54	18	Unpublished
	U41	4.79263	66.38	NZ_CP015732.1	3	4,407	4,134	51	15	Unpublished
	DCT-5	4.53075	66.22	NZ_CP029642.1	1	4,040	3,816	50	15	Unpublished
	PGP41	4.27024	65.40	NZ_CP026514.1	0	3,917	3,760	49	12	Unpublished
	ERGS1:01	4.93669	65.41	NZ_CP012479.1	2	4,481	4,232	41	6	[36]
	YC-RL1	4.01864	64.04	NZ_CP013297.1	2	3,754	3,606	66	19	[37]
	Hiyo4	3.77925	65.00	AP014718.1	0	5,182	5,120	50	12	[38]
	KBS0702	3.64955	67.90	NZ_CP042172.1	0	3,373	3,243	51	15	Unpublished
	UKPF54-2	3.51782	68.50	NZ_CP040174.1	0	3,238	3,110	50	15	Unpublished
	MN05-02	3.64342	68.81	AP018697.1	1	3,608	3,543	52	12	Unpublished
	Hiyo8	5.02672	63.76	AP014719.1	2	7,108	7,038	53	15	[38]
	ATCC21022	4.43490	63.40	NZ_CP014196.1	0	4,078	3,910	53	12	[39]
*Arthrobacter crystallopoietes*	DSM 20117	5.03270	64.36	NZ_CP018863.1	2	4,634	4,425	48	15	Unpublished
*Arthrobacter alpinus*	R3.8	4.04645	62.20	NZ_CP012677.1	0	3,732	3,523	51	18	Unpublished
	ERGS4:06	4.33365	60.59	NZ_CP013200.1	1	3,850	3,581	53	25	[40]
	A3	4.45829	60.64	NZ_CP013745.1	1	4,033	3,902	52	19	Unpublished
*Pseudarthrobacter phenanthrenivorans*	Sphe3	4.53532	65.38	NC_015145.1/CP002379.1	2	4,278	4,052	50	12	[41]
*Pseudarthrobacter chlorophenolicus*	A6	4.98087	65.98	NC_011886.1/CP001341.1	2	4,685	4,505	49	15	[42]
*Pseudarthrobacter sulfonivorans*	Ar51	5.04376	64.70	NZ_CP013747.1	1	4,640	4,408	50	12	Unpublished

### Bacterial glycogen metabolism in a cold environment

**Fig. 5:**
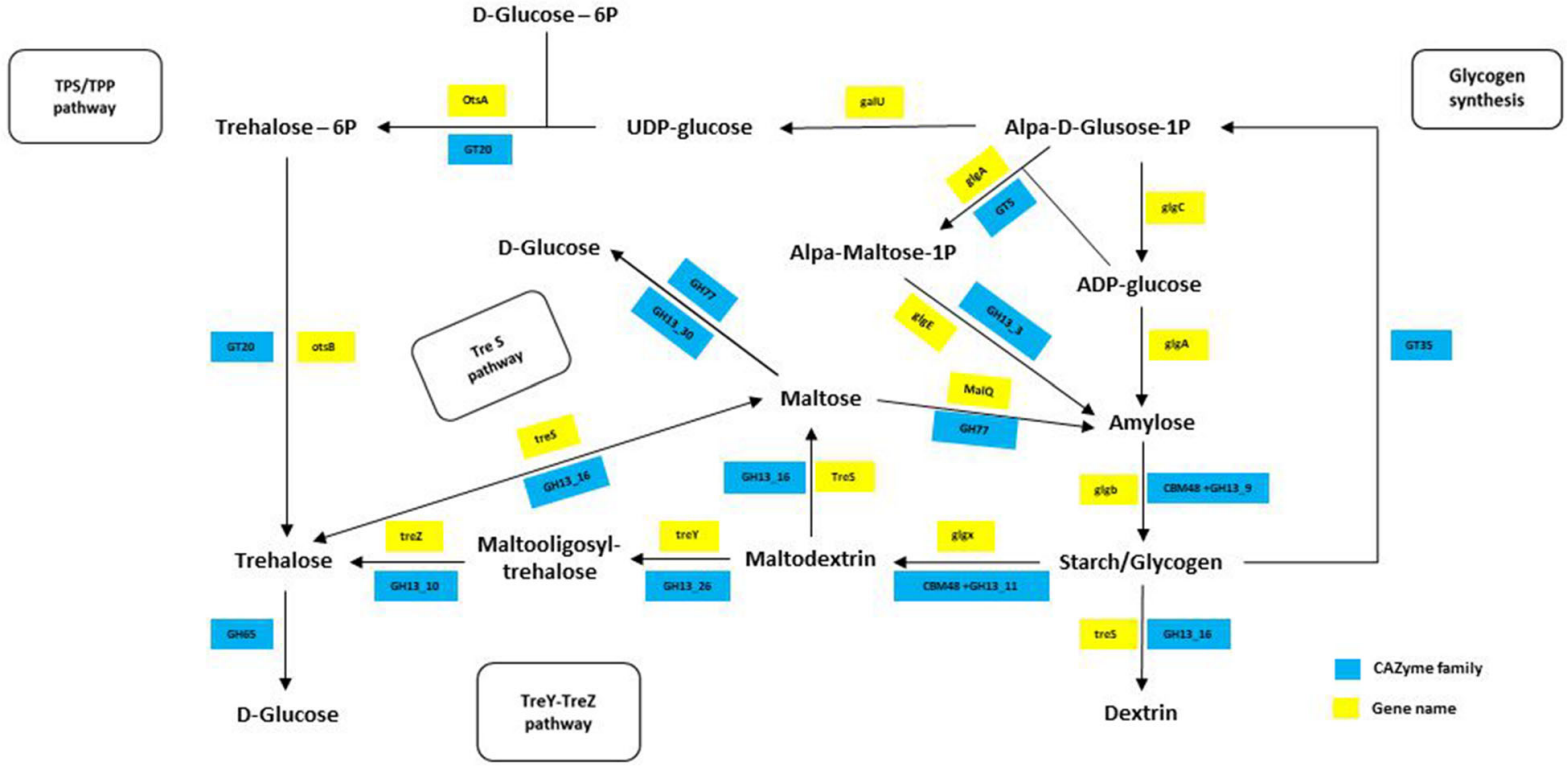
Predicted pathways for glycogen and trehalose metabolism in *Arthrobacter* sp. PAMC25564 as cold adaptation response. Kyoto Encyclopedia of Genes and Genomes KEGG-predicted enzyme pathways (yellow squares), and dbCAN2-predicted CAZyme family (blue squares)

Glycogen is an energy source for plants, animals, and bacteria and is one of the most common carbohydrates. Glycogen consists of D-glucose residues joined by α (1→4) links; and it is a structural part of cellulose and dextran [43]. Glycogen is a polymer with approximately 95 % of α-1, 4 linkages, and 5 % of α-1, 6 branching linkages. In bacteria, glycogen metabolism includes five essential enzymes: ADP-glucose pyrophosphorylase (GlgC), glycogen synthase (GlgA), glycogen branching enzyme (GlgB), glycogen phosphorylase (GlgP), and glycogen debranching enzyme (GlgX) [44]. To adapt and survive in a cold environment, organisms need well-developed functional energy storage systems, one of which is glycogen synthesis. Bacteria have a passive energy saving strategy to adapt to cold environmental conditions such as nutrient deprivation, by using slow glycogen degradation. Glycogen is hypothesized to function as long durability energy reserves, which have been reported as a Durable Energy Storage Mechanism (DESM) to account for the long-term survival of some bacteria in cold environments [45]. Metabolism of maltodextrin has been linked with osmoregulation and sensitivity of bacterial endogenous induction to hyperosmolarity, which is related to glycogen metabolism. Glycogen-generated maltotetraose is dynamically metabolized by maltodextrin phosphorylase (MalP) and maltodextrin glucosidase (MalZ), while 4-α-glucanotransferase (MalQ) is responsible for maltose recycling to maltodextrins [46]. Maltotetraose is produced using GlgB, MalZ, MalQ, and glucokinase (Glk), which act on maltodextrin and glucose. On the other hand, glucose-1-phosphate can be formed by MalP for glycogen synthesis or glycolysis [47]. Thus, glycogen degradation can play an essential role in bacterial adaptation to the environment. Additionally, maltose may form capsular α-glucan, which plays a role in environmental adaptation through the (TreS)-Pep2-GlgE-GlgB pathway [48, 49]. Previous studies indicate that trehalose is involved in bacterial adaptation to temperature fluctuation, hyperosmolarity, and desiccation resistance. Recently, the accumulation of trehalose and glycogen under cold conditions in *Propionibacterium freudenreichii* has been reported [50, 51]. Therefore, the role of glycogen in bacterial energy metabolism is closely linked to several metabolic pathways associated with bacterial persistence under environmental stresses such as starvation, drying, temperature fluctuations, and hyperosmolarity. Maltodextrin and trehalose pathways are examples of the relationship between glycogen and other metabolic pathways, as shown in Fig. 5. However, further exploration is needed to elucidate the relationship of glycogen with other compounds, and the mechanisms involved in bacterial persistence strategies [46]. The comparative analysis of predicted pathways for glycogen metabolism in *Arthrobacter* isolates (Additional file 2: Table S1), showed that in PAMC25564 the trehalose biosynthesis follows three metabolic pathways (OtsAB, TreYZ, and TreS) as in *Mycobacterium* [52]. The trehalose biosynthesis pathway is well known in numerous bacteria, for example, as a defense strategy involving the accumulation of trehalose. Three metabolic pathways to regulate osmotic stress have been reported in *Corynebacterium glutamicum* [53]. These three metabolic pathways are used for producing trehalose in *C. glutamicum*, where the gene *galU*/*otsAB* allows the increase of trehalose levels up to six times [54, 55]. This pathway was found in *A.* sp. PAMC25564, and it was predicted that such an isolate could produce energy in cold environments.

### Glycogen metabolism and the trehalose pathway in Arthrobacter species

**Fig. 6:**
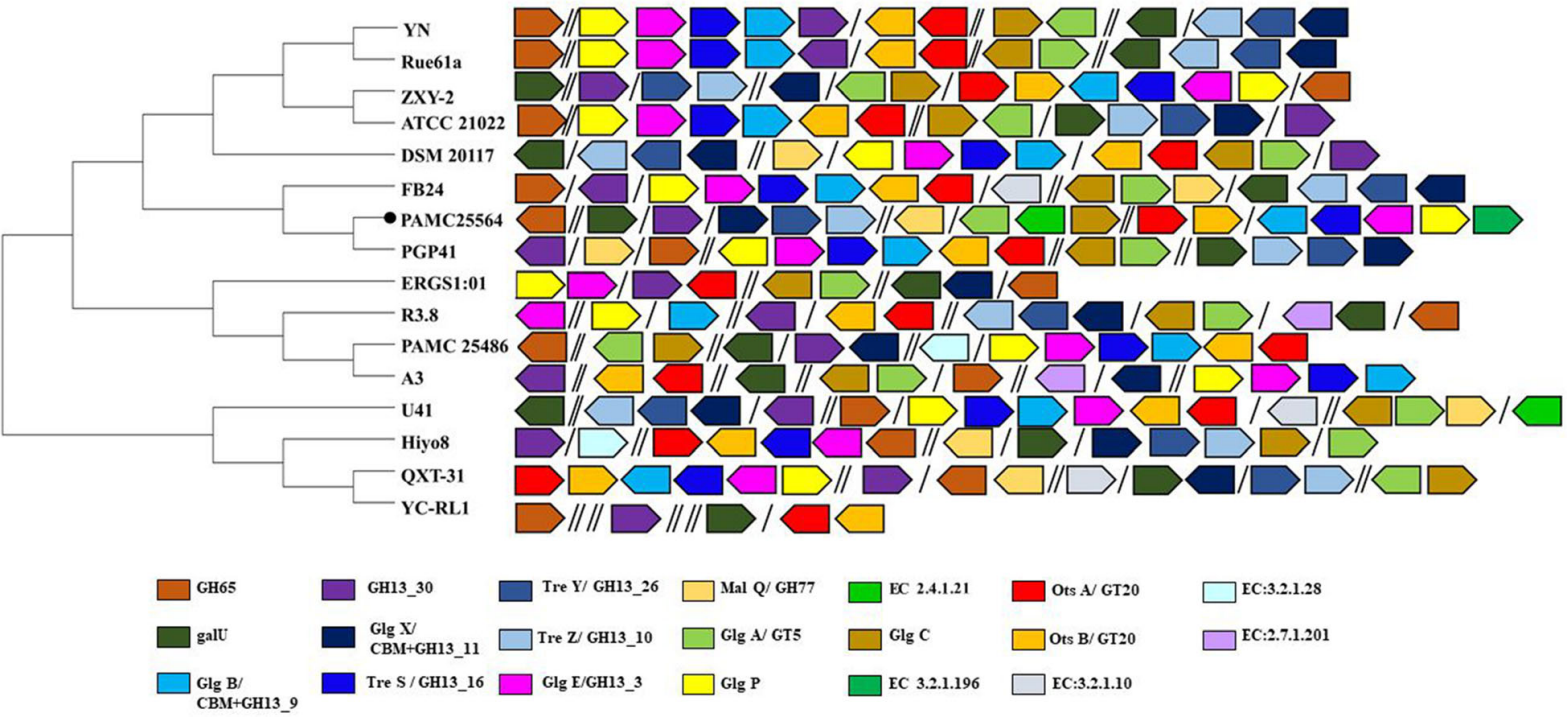
Comparative analysis of predicted glycogen and trehalose metabolic pathways in *Arthrobacter* species. Glycogen and trehalose metabolism-associated genes are colored, as indicated below the figure. The figure shows the differences in direction, presence, and location of genes among the strains. GH65: α,α-trehalose phosphorylase; GH13_30: α-1,4-glucan-maltose-1-phosphate maltosyltransferase; TreY/GH13_26: 1→4;-α-D-glucan 1-α-D-glucosylmutase; MalQ: 4-α-glucanotransferase; 2.4.1.21: glycogen synthase, and ADP-glucose transglucosylase; OtsA/ GT20: trehalose 6-phosphate synthase; 3.2.1.28: α-trehalase; GalU: UTP-glucose-1-phosphate uridylyltransferase; GlgX/CBM + GH13_11: glycogen debranching protein; TreZ/GH13_10: malto-oligosyltrehalose trehalohydrolase, α-glucosidase, and α-trehalase; GlgA/GT20: glycogen synthase, and ADP-glucose transglucosylase; GlgC: glucose-1-phosphate adenylyltransferase; OtsB/GT20: trehalose 6-phosphate phosphatase; 2.7.1.201: PTS system, and sugar-specific IIA component; GlgB/CBM + GH13_9: 1,4-α-glucan branching enzyme; TreS/GH13_16: maltose α-D-glucosyltransferase/α-amylase; GlgE/GH13_3: α-1,4-glucan-maltose-1-phosphate maltosyltransferase, PTS system, and sugar-specific IIA component; GlgP: glycogen phosphorylase; and 3.2.1.10: oligo-1,6-glucosidase. The black circle indicates our strain. / mean one gene deletion and // mean two more than genes deletion

We investigated the glycogen metabolic pathways in each *Arthrobacter* species strain (Fig. 6). To determine the three pathways of glycogen metabolism and trehalose biosynthesis in *Arthrobacter* species coming from diverse environments, the level of dissimilarity was analyzed based on the composition of GH, GT, and other major enzymes from the 16 genomes. The analysis showed that only QXT-31, U41, and PGP41 shared the same genes and pathway as our strain, while other strains have a slightly different pattern. This study assumed that the PAMC25564 strain uses different pathways to obtain energy or degrade polysaccharides. Based on the above-mentioned pathway-related genes, we confirmed that strains YN, Rue61, PAMC25486, ZXY-2, ERGS1:01, YC-RL1, ATCC21022, R.3.8, and A3 lack the *malQ* gene. These strains of *Arthrobacter* species showed a low number of genes for the three main pathways of trehalose biosynthesis and these are responsible for maltose recycling to maltodextrins. Therefore, the energy supply may be compromised in such isolates. Although most strains showed *galU/otsAB* genes, strains Hyo8 and ERGS1:01 lack the *otsB* gene (Fig. 6; Additional file 2: Table S1). This result suggests that these strains would produce a significantly lower amount of trehalose than the isolates having the *otsB* gene. Additionally, we investigated the phosphotransferase system-related genes in strains R.3.8 and A3. These enzymes constitute another method used by bacteria for sugar uptake when the source of energy is phosphoenolpyruvate. As a result, these two strains probably produce polysaccharides by themselves or from an external source using phosphoenolpyruvate rather than consuming energy. Most of the compared strains were isolated in low-temperature (-18 to 15 ℃) and low-contamination environments. These species have been reported as highly anticipated strains due to their fast adaptability to the environment, and these results suggest this adaptability is related to glycogen metabolism, trehalose, and maltodextrin pathways, which may have an impact on industrial applications. Microorganisms with related genes can make trehalose production economical and are able to draw on their own energy. This result also predicted that trehalose metabolism in microorganisms depends on the requirement of bacterial metabolism in the given environmental conditions, and this is one of the characteristics of bacteria that grow in extreme environments such as the cold.

## Conclusions

In this study, we elucidated the complete genome sequence of *Arthrobacter* sp. PAMC25564 and conducted a comparative genome analysis with other species for studying CAZyme patterns. We isolated bacteria from cryoconite under laboratory conditions and confirmed that the isolate is an *Arthrobacter* species, based on the analysis of 16 S rRNA sequences. Although the isolation of this species from extreme or contaminated environments has been reported previously, there are no reports on the use of CAZymes in cold environments. We predicted that *Arthrobacter* sp. PAMC25564 could produce energy autonomously as fast as it can adapt to the environment. The PAMC25564 strain genome is 4.17 Mb in size with a GC content of 66.74 %. The analysis of its complete genome suggested that the isolate has glycogen, trehalose, and maltodextrin pathways associated to CAZyme genes. We confirmed that PAMC25564 has 108 active CAZyme genes from the following groups, 5 AA, 2 CBM, 23 CE, 33 GH, and 45 GT. In addition, a comparative genome analysis of *Arthrobacter* species revealed that they adapt quickly to the environment. In conclusion, we expect the genome sequence analysis to provide valuable information regarding novel functional enzymes, especially CAZymes, which are active at low temperatures and can be used for biotechnological applications and fundamental research purposes. This study provides a foundation to understand how the PAMC25564 strain produces energy in an extreme environment.

## Methods

### Isolation and genomic DNA extraction of Arthrobacter sp. PAMC25564

The strain *Arthrobacter* sp. PAMC25564 was isolated from the Cryoconite of Wurmkogel, Ötztaler Alps, Austria (47°04’ N, 12°41’ E, 2820 m to a maximum elevation) using 0.1 X R2A agar (MB cell Ltd., Seoul, Korea). The strain was isolated at an environmental temperature of 20 ℃. The bacterial sample for DNA analysis was isolated at 15 ℃ by using a pure R2A agar. DNA from *A.* sp. PAMC25564 was extracted using a QIAamp DNA Mini Kit (Qiagen Inc., Valencia, CA, USA). The quantity and purity of genomic DNA were determined using a spectrophotometer (Biochrome, Libra S35PC, UK). The extracted DNA was checked by agarose gel electrophoresis to evaluate its quality. DNA was stored at -20 ℃ until use.

### Genome sequencing and assembly of the whole genome of Arthrobacter sp. PAMC25564

Genome sequencing was performed using PacBio sequel single-molecule real-time (SMRT) sequencing technology (Pacific Biosciences, Menlo Park, CA, USA). SMRTbell library inserts (20 kb) were sequenced using SMRT cells. Raw sequence data were generated from 7,698 reads and 4,170,970 bp that were assembled de novo by using the hierarchical genome-assembly process (HGAP v.4) protocol [56] and HGAP4 assembly using SMRT analysis software (ver. 2.3; Pacific Biosciences, https://github.com/PacificBiosciences/SMRT-Analysis). The complete genome sequence was deposited in the GenBank database under the GenBank accession number NZ_CP039290.1.

### Genome annotation of Arthrobacter sp. PAMC25564

The genome of strain PAMC25564 was annotated using the rapid annotation subsystem technology (RAST) server [57]. The predicted gene sequences were translated and searched in the National Center for Biotechnology Information (NCBI) non-redundant database, the COG from the eggnog v.4.5.1 database [58], and the Kyoto Encyclopedia of Genes and Genomes (KEGG) database by cutoff value 0.01 [59]. A circular map of the PAMC25564 genome was prepared using the CGView comparison tool [60]. CAZyme gene analyses were carried out by running dbCAN tool [61] scans using hidden Markov model (HMM) profile downloaded from dbCAN2 HMMdb (version 7.0). At the same time, we obtained results from Signal P (version 4.0) about the presence of CAZyme genes [62]. The e-value cutoff was 1e-15 and the coverage cutoff was > 0.35. In addition, we used DIAMOND [63] (e-value < 1e102) and Hotpep [64] (frequency > 2.6, hits > 6) to improve the prediction accuracy.

### Phylogenetic analysis

Strain PAMC25564 was compared with other *Arthrobacter* species using 16 S rRNA phylogenetic analysis. Alignments were performed using Basic Local Alignment Search Tool from the NCBI database and analyzed using EzBio Cloud (www.ezbiocloud.com). 16 S rRNA sequences were aligned using MUSCLE [65, 66] and MEGA X [67] to reconstruct a neighbor-joining tree and maximum likelihood tree with 1,000 bootstrap replications.

### Comparative genomics of Arthrobacter sp. PAMC25564

We used all complete genome sequences of *Arthrobacter* species available in GenBank (https://www.ncbi.nlm.nih.gov). Firstly, we determined the relationship of PAMC25564 with other strains from the same species using complete genome sequences and checked their similarity by comparing values of ANI, calculated using an OrthoANI in OAT (the Orthologous Average Nucleotide Identity Tool) [68]. The genome information of several *Arthrobacter* species is available in GenBank, and we compared the CAZymes from registered species referenced in CAZy (http://www.cazy.org). First, based on the 16 S rRNA sequence, strains with similarity were selected, and then 25 strains with a complete genome were chosen. All those sequences were downloaded from the database and all CAZymes were reannotated using the dbCAN2 server. In fact, some kinds of strains (PAMC 25,486, ERGS1:01, and ERGS4:06) reported isolated from cold environments such as the Spitsbergen of Arctic, glacial of Himalaya.

## Supplementary Information

**Additional file 1: Supplementary Figure S1**. Comparative CAZyme-encoding genes found in the genome of Arthrobacter species. GT, glycosyl transferase; GH, glycoside hydrolase; CE, carbohydrate esterase; CBM, carbohydrate binding; and AA, auxiliary activity. CAZyme-encoding genes are colored, as indicated below the figure. Arthrobacter sp.: PAMC25564, 24S4-2, YN, QXT-31, Rue61a, FB24, PAMC25486, ZXY-2, U41, DCT-5, PGP41, ERGS1:01, YC-RL1, Hiyo4, KBS0702, UKPF54-2, MN05-02, Hiyo8, and ATCC21022; Arthrobacter crystallopoietes: DSM 20117; Arthrobacter alpinus: R3.8, ERGS4:06, and A3; Pseudarthrobacter phenanthrenivorans: Sphe3; Pseudarthrobacter chlorophenolicus: A6; and Pseudarthrobacter sulfonivorans: Ar51.

**Additional file 2: Supplementary Table 1.** Comparative analysis of predicted pathways for glycogen and trehalose metabolism in Arthrobacter species. The symbol + indicates that the isolate produces the enzyme but symbol - indicates that the not produces the enzyme.

## Data Availability

The datasets analyzed during the current study are available in the NCBI repository, accession numbers: NZ_CP039290.1for *Arthrobacter* sp. strain PAMC25564, complete genome; NZ_CP040018.1 for *Arthrobacter* sp. strain 24S4-2, complete genome; NZ_CP022436.1 for *Arthrobacter* sp. strain YN, complete genome; NZ_CP019304.1 for *Arthrobacter* sp. strain QXT-31, complete genome; NZ_CP003203.1 for *Arthrobacter* sp. strain Rue61a; NZ_CP000454.1 for *Arthrobacter* sp. strain FB24, complete genome; NZ_CP007595.1 for Arthrobacter sp. strain PAMC25486, complete genome; NZ_CP017421.1 for *Arthrobacter* sp. strain ZXY-2, complete genome; NZ_CP015732.1 for *Arthrobacter* sp. strain U41, complete genome; NZ_CP029642.1 for *Arthrobacter* sp. strain DCT-5, complete genome; NZ_CP026514.1 for *Arthrobacter* sp. strain PGP41, complete genome; NZ_CP012479.1 for *Arthrobacter* sp. strain ERGS1:01, complete genome; NZ_CP013297.1 for *Arthrobacter* sp. strain YC-RL1, complete genome; AP014718.1 for *Arthrobacter* sp. strain Hiyo4, complete genome; NZ_CP042172.1 for *Arthrobacter* sp. strain KBS0702, complete genome; NZ_CP040174.1 for *Arthrobacter* sp. strain UKPF54-2, complete genome; AP018697.1 for *Arthrobacter* sp. strain MN05-02, complete genome; AP014719.1 for *Arthrobacter* sp. strain Hiyo8, complete genome; NZ_CP014196.1 for *Arthrobacter* sp. strain ATCC21022, complete genome. NZ_CP018863.1 for *Arthrobacter crystallopoietes* DSM 20117, complete genome; NZ_CP012677.1 for *Arthrobacter alpinus* strain R3.8, complete genome; NZ_CP013200.1 for *Arthrobacter alpinus* strain ERGS4:06, complete genome; NZ_CP013745.1 for *Arthrobacter alpinus* strain A3, complete genome; CP002379.1 for *Pseudarthrobacter phenanthrenivorans* strain Sphe3, complete genome; CP001341.1 for *Pseudarthrobacter chlorophenolicus* strain A6, complete genome; NZ_CP013747.1 for *Pseudarthrobacter sulfonivorans* strain Ar51, complete genome.

## Abbreviations

GH: Glycoside Hydrolase
CE: Carbohydrate Esterase
PL: Polysaccharide Lyase
GT: Glycosyltransferase
AA: Auxiliary Activity
CBM: Carbohydrate-Binding Module
ANI: Average Nucleotide Identity
NCBI: National Center for Biotechnology Information
