# Supplementary material for: Complete genome sequence of Arthrobacter sp. PAMC25564 and its comparative genome analysis for elucidating the role of CAZymes in cold adaptation
Source: BMC Genomics. 2021 Jun 2;22:403. doi: 10.1186/s12864-021-07734-8 (PMC8171050; doi:10.1186/s12864-021-07734-8)
Supplement: Supplementary file 1 — Additional file 1: Supplementary Figure S1. Comparative CAZyme-encoding genes found in the genome of Arthrobacter species. GT, glycosyl transferase; GH, glycoside hydrolase; CE, carbohydrate esterase; CBM, carbohydrate binding; and AA, auxiliary activity. CAZyme-encoding genes are colored, as indicated below the figure. Arthrobacter sp.: PAMC25564, 24S4-2, YN, QXT-31, Rue61a, FB24, PAMC25486, ZXY-2, U41, DCT-5, PGP41, ERGS1:01, YC-RL1, Hiyo4, KBS0702, UKPF54-2, MN05-02, Hiyo8, and ATCC21022; Arthrobacter crystallopoietes: DSM 20117; Arthrobacter alpinus: R3.8, ERGS4:06, and A3; Pseudarthrobacter phenanthrenivorans: Sphe3; Pseudarthrobacter chlorophenolicus: A6; and Pseudarthrobacter sulfonivorans: Ar51. [file 12864_2021_7734_MOESM1_ESM.docx]

**Complete genome sequence of *Arthrobacter* sp. PAMC25564 and its comparative genome analysis for elucidating the role of CAZymes in cold adaptation**

So-Ra Han^1^, Byeollee Kim^1^, Jong Hwa Jang^2^, Hyun Park^3,*^, and Tae-Jin Oh^1, 4, 5,*^

^1^ Department of Life Science and Biochemical Engineering, Graduate School, SunMoon University, 70 Sunmoon-ro 221, Tangjeong-myeon, Asan-si, Chungnam 31460, Republic of Korea

^2^ Department of Dental Hygiene, College of Health Science, Dankook University, 119 Dandae-ro, Dongnam-gu, Cheonan-si, Chungnam 31116, Republic of Korea

^3^ Division of Biotechnology, College of Life Science and Biotechnology, Korea University, Seoul 02841, Republic of Korea

^4^ Genome-based BioIT Convergence Institute, 70 Sunmoon-ro 221, Tangjeong-myeon, Asan-si, Chungnam 31460, Republic of Korea

^5^ Department of Pharmaceutical Engineering and Biotechnology, SunMoon University, 70 Sunmoon-ro 221, Tangjeong-myeon, Asan-si, Chungnam 31460, Republic of Korea

* Corresponding authors: Hyun Park & Tae-Jin Oh

H. Park, Division of Biotechnology, College of Life Sciences and Biotechnology, Korea University, Seoul 02841, Korea. Tel: +82 2 3290 3051; E-mail: hpark@korea.ac.kr

T.-J. Oh, Department of Pharmaceutical Engineering and Biotechnology, SunMoon University, Asan 31460, Korea. Tel: +82 41 530 2677; E-mail: [tjoh3782@sunmoon.ac.kr](mailto:tjoh3782@sunmoon.ac.kr)

**
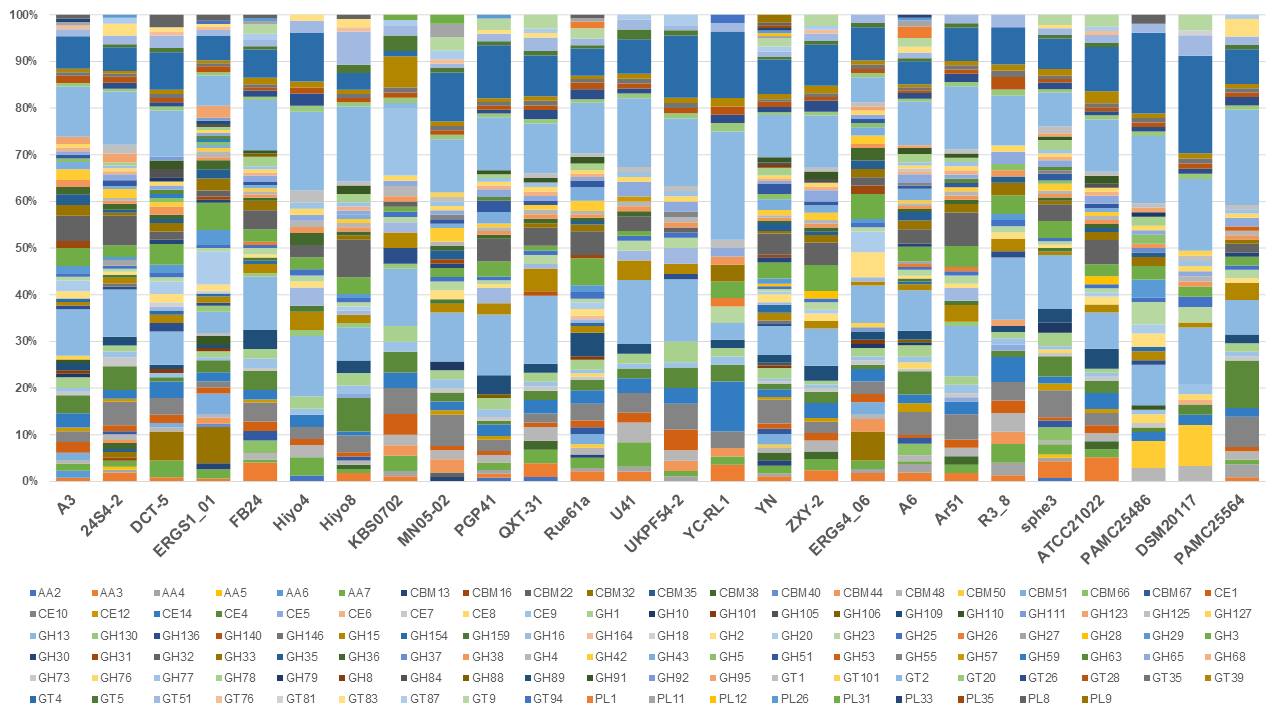
**

**Figure S1.** **Comparative CAZyme-encoding genes found in the genome of *Arthrobacter* species.** GT, glycosyl transferase; GH, glycoside hydrolase; CE, carbohydrate esterase; CBM, carbohydrate binding; and AA, auxiliary activities. CAZyme-encoding genes are colored as indicated below the figure. *Arthrobacter* sp.: PAMC25564, 24S4-2, YN, QXT-31, Rue61a, FB24, PAMC25486, ZXY-2, U41, DCT-5, PGP41, ERGS1:01, YC-RL1, Hiyo4, KBS0702, UKPF54-2, MN05-02, Hiyo8, and ATCC21022; *Arthrobacter crystallopoietes*: DSM 20117; *Arthrobacter alpinus*: R3.8, ERGS4:06, and A3; *Pseudarthrobacter phenanthrenivorans*: Sphe3; *Pseudarthrobacter chlorophenolicus*: A6; and *Pseudarthrobacter sulfonivorans*: Ar51.
