# Supplementary material for: Complete genome sequence of Arthrobacter sp. PAMC25564 and its comparative genome analysis for elucidating the role of CAZymes in cold adaptation
Source: BMC Genomics. 2021 Jun 2;22:403. doi: 10.1186/s12864-021-07734-8 (PMC8171050; doi:10.1186/s12864-021-07734-8)
Supplement: Supplementary file 2 — Additional file 2: Supplementary Table 1. Comparative analysis of predicted pathways for glycogen and trehalose metabolism in Arthrobacter species. The symbol + indicates that the isolate produces the enzyme but symbol - indicates that the not produces the enzyme. [file 12864_2021_7734_MOESM2_ESM.docx]

**Complete genome sequence of *Arthrobacter* sp. PAMC25564 and its comparative genome analysis for elucidating the role of CAZymes in cold adaptation**

So-Ra Han^1^, Byeollee Kim^1^, Jong Hwa Jang^2^, Hyun Park^3,*^, and Tae-Jin Oh^1, 4, 5,*^

^1^ Department of Life Science and Biochemical Engineering, Graduate School, SunMoon University, 70 Sunmoon-ro 221, Tangjeong-myeon, Asan-si, Chungnam 31460, Republic of Korea

^2^ Department of Dental Hygiene, College of Health Science, Dankook University, 119 Dandae-ro, Dongnam-gu, Cheonan-si, Chungnam 31116, Republic of Korea

^3^ Division of Biotechnology, College of Life Science and Biotechnology, Korea University, Seoul 02841, Republic of Korea

^4^ Genome-based BioIT Convergence Institute, 70 Sunmoon-ro 221, Tangjeong-myeon, Asan-si, Chungnam 31460, Republic of Korea

^5^ Department of Pharmaceutical Engineering and Biotechnology, SunMoon University, 70 Sunmoon-ro 221, Tangjeong-myeon, Asan-si, Chungnam 31460, Republic of Korea

* Corresponding authors: Hyun Park & Tae-Jin Oh

H. Park, Division of Biotechnology, College of Life Sciences and Biotechnology, Korea University, Seoul 02841, Korea. Tel: +82 2 3290 3051; E-mail: hpark@korea.ac.kr

T.-J. Oh, Department of Pharmaceutical Engineering and Biotechnology, SunMoon University, Asan 31460, Korea. Tel: +82 41 530 2677; E-mail: [tjoh3782@sunmoon.ac.kr](mailto:tjoh3782@sunmoon.ac.kr)

**Supplementary Table S1. Comparative analysis of predicted pathways for glycogen and trehalose metabolism in *Arthrobacter* species.**

| **Enzyme** | **CAZyme** | **EC number** | **Functions** | **PAMC**  **25564** | **YN** | **QXT-31** | **Rue61a** | **FB24** | **PAMC 25486** | **ZXY-2** | **U41** | **PGP41** | **ERGS1:01** | **YC-RL1** | **Hiyo8** | **ATCC21022** | **DSM 20117** | **R3.8** | **A3** |
| --- | --- | --- | --- | --- | --- | --- | --- | --- | --- | --- | --- | --- | --- | --- | --- | --- | --- | --- | --- |
| GlgP | GT35 | 2.4.1.1 | glycogen phosphorylase | + | + | + | + | + | + | + | + | + | + | **-** | + | + | + | + | + |
| OtsA | GT20 | 2.4.1.15 | trehalose 6-phosphate synthase | + | + | + | + | + | + | + | + | + | + | + | + | + | + | + | + |
| Glgb | CBM48+GH13_9 | 2.4.1.18 | 1,4-α-glucan branching enzyme | + | + | + | + | + | + | + | + | + | **-** | **-** | + | + | + | + | + |
| GlgA |  | 2.4.1.21 | glycogen synthase, ADP-glucose transglucosylase | + | **-** | + | + | + | + | + | + | + | + | **-** | + | + | + | + | + |
| MalQ | GH77 | 2.4.1.25 | 4-α-glucanotransferase | + | **-** | + | **-** | + | **-** | **-** | + | + | **-** | **-** | + | **-** | + | **-** | **-** |
| GlgA | GT5 | 2.4.1.342 | α-maltose-1-phosphate synthase | + | + | + | + | + | + | + | + | + | + | **-** | + | + | + | + | + |
|  | GH65 | 2.4.1.64 | α,α-trehalose phosphorylase | + | + | + | + | + | + | + | + | + | + | + | + | + | **-** | + | + |
| GlgE | GH13_30 | 2.4.99.16 | α-1,4-glucan-maltose-1-phosphate maltosyltransferase | + | + | + | + | + | + | + | + | + | + | **-** | + | + | + | + | + |
|  |  | 2.7.1.201 | PTS system, sugar-specific IIA component | **-** | **-** | **-** | **-** | **-** | **-** | **-** | **-** | **-** | **-** | **-** | **-** | **-** | **-** | + | + |
| GlgC |  | 2.7.7.27 | glucose-1-phosphate adenylyltransferase | + | + | + | + | + | + | + | + | + | + | **-** | + | + | + | + | + |
| GalU |  | 2.7.7.9 | UTP-glucose-1-phosphate uridylyltransferase | + | + | + | + | + | + | + | + | + | + | + | + | + | + | + | + |
| OtsB | GT20 | 3.1.3.12 | trehalose 6-phosphate phosphatase | + | + | + | + | + | + | + | + | + | **-** | + | **-** | + | + | + | + |
| TreS | GH13_16 | 3.2.1.1 | maltose α-D-glucosyltransferase / α-amylase | + | + | + | + | + | + | + | + | + | **-** | **-** | + | + | + | **-** | + |
|  |  | 3.2.1.10 | oligo-1,6-glucosidase | **-** | **-** | + | **-** | + | **-** | **-** | + | **-** | **-** | **-** | **-** | **-** | **-** | **-** | **-** |
| TreZ | GH13_10 | 3.2.1.141 | maltooligosyltrehalose trehalohydrolase | + | + | + | + | + | **-** | + | + | + | **-** | **-** | + | + | + | + | **-** |
|  | GH13_30 | 3.2.1.20 | α-glucosidase | + | + | + | + | + | + | + | + | + | + | + | + | + | + | + | + |
|  |  | 3.2.1.28 | α-trehalase | **-** | **-** | **-** | **-** | **-** | + | **-** | **-** | **-** | **-** | **-** | + | **-** | **-** | **-** | **-** |
| GlgX | CBM48+GH13_11 | 3.2.1.68 | glycogen debranching protein | + | + | + | + | + | + | + | + | + | + | **-** | + | + | + | + | + |
| TreY | GH13_26 | 5.4.99.15 | (1→4)-α-D-glucan 1-α-D-glucosylmutase | + | + | + | + | + | **-** | + | + | + | **-** | **-** | + | + | + | + | **-** |
| TreS | GH13_16 | 5.4.99.16 | maltose α-D-glucosyltransferase / α-amylase | + | + | + | + | + | + | + | + | + | **-** | **-** | + | + | + | **-** | + |

The symbol + indicates that the isolate produces the enzyme but symbol - indicates that the not produces the enzyme.
